# Alteration of physical activity during COVID-19 pandemic lockdown in young adults

**DOI:** 10.1186/s12967-020-02591-7

**Published:** 2020-11-02

**Authors:** Bruno C. Huber, Julius Steffen, Jenny Schlichtiger, Tanja Graupe, Eileen Deuster, Victoria P. Strouvelle, Martin R. Fischer, Steffen Massberg, Stefan Brunner

**Affiliations:** 1 Department of Medicine I, Ludwig-Maximilians-University Munich, University Hospital Munich – Campus Innenstadt, Ziemssenstrasse 1, 80336 Munich, Germany; 2 DZHK (German Centre for Cardiovascular Research), Munich Heart Alliance (MHA), Partner Site Munich, Munich, Germany; 3 Institute for Medical Education, University Hospital, LMU Munich, Munich, Germany

**Letter to the Editor,**

In December 2019, the new severe acute respiratory syndrome coronavirus 2 (SARS-CoV-2), emerged in China and rapidly spread throughout the world [1]. In order to flatten the curve of exponential growth, many countries imposed a lockdown. In Bavaria, exceptions to the curfew were going to work, necessary shopping, visits to doctors and pharmacies, assisting others, visits from partners—and also exercise outside, but only alone or with other household members. Despite efforts to allow people to stay physically active, many opportunities, such as fitness centres, athletic programs and sports clubs, have been suspended.

Physical activity has a beneficial effect on many risk factors, and reduces the risk of mortality in a dose-dependent manner. Vice versa, a sedentary lifestyle is a significant risk factor for chronic diseases, and mortality [2].

To the best of our knowledge, the effect of pandemics on physical activity has not been investigated yet. However, a previous study has assessed the impact of natural disasters on physical activity. After the devastating earthquake and tsunami in Japan in 2011, there was a significant decrease in physical activity detectable over the three years following the disaster [3].

In our study, we aimed to determine the impact of the COVID-19 pandemic lockdown on physical activity in young adults.

This trial is a cross-sectional study, which was performed in accordance with the Declaration of Helsinki. It was approved by the ethics committee of the Ludwig-Maximilians-University (LMU) Munich, Germany (approval number 20–268 KB).

In order to analyse physical activity during lockdown measures compared to a typical week before, we conducted an online survey, which was distributed via email among students of major Bavarian universities. Among others, step count data from smartphones or wearables should be provided, if available. Three consecutive days (Sunday, Monday, and Tuesday) were chosen to represent the periods before, and after, lockdown (19^th^, 20^th^, 21^st^ of January and 22^nd^, 23^rd^, 24^th^ of March).

Statistical analyses were performed using SPSS version 25. For the description of data, absolute and relative frequencies were calculated as well as measures of central tendency (mean, median) and dispersion (min, max, standard deviation). Categorical variables were compared using the Chi-squared test. Normal distribution was tested using the Kolmogorov–Smirnov test. To calculate differences in the mean step count, Wilcoxon signed-rank test and Mann–Whitney U test were used. Statistical significance was determined at p < 0.05.

A total of 1980 students at six different Bavarian universities took part in the large-scale online survey (response rate, 24%). The mean age was 23.3 ± 4.0 years (mean ± standard deviation), 71.5% (n = 1371) were female, and the majority of participants had a normal body mass index (BMI) (22.1 ± 4.5 kg/m^2^). A SARS-CoV-2 swab was performed in 6.6% (n = 127) of the participants, and, in total, 0.4% (n = 7) had a positive result.

The implementation of lockdown led to a decrease of physical activity in 44.5% (n = 867) of the participants, 32.8% (n = 639) reported an increased amount of training (Fig. 1a). Participants were asked to semi-quantify the amount of physical activity on a 4-level scale. More than 50% stated to have been exercising 2–5 h weekly before lockdown. Afterwards, only 39.7% reported likewise, and the amount of participants doing 0 or up to 2 exercise hours per week increased. Further, after lockdown, more people (24.0 after vs. 20.2% before lockdown) stated to do more than 5 h per week (Fig. 1b). Interestingly, 76% of the formerly inactive group has now increased their physical activity significantly.

**Fig. 1:**
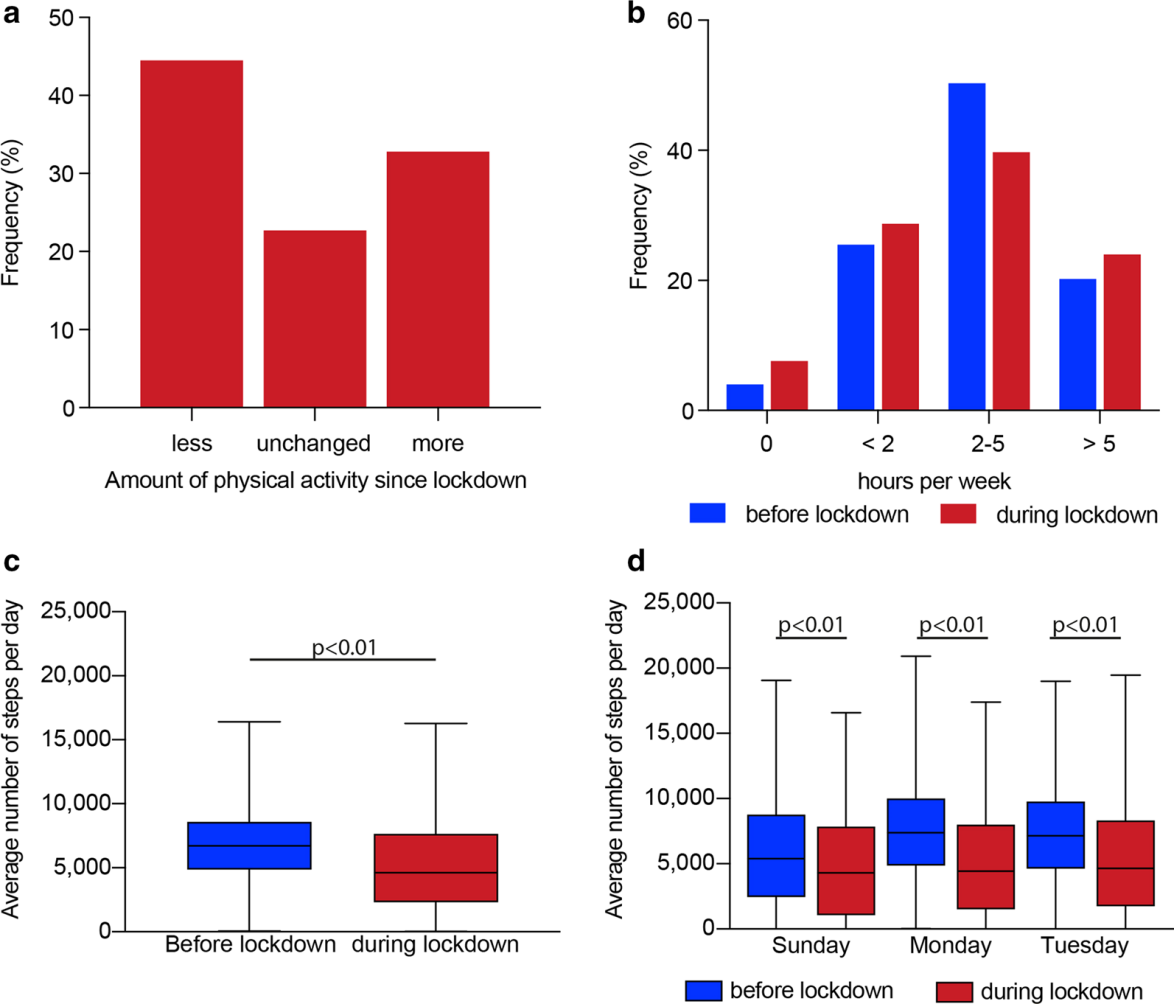
Physical activity before and after lockdown: **a** Participants were asked if the amount of physical activity had changed after the lockdown. 44.5% (867) reported to be less active, and 32.8% (639) reported to be more active than before the lockdown. In 22.7% (443) of the participants, the amount of physical activity was unchanged. **b** Participants were further asked to semi-quantify the average hours per week of physical activity they had done in a typical week before, and after, the lockdown. The self-assessment of hours of physical activity per week revealed a marked decrease in the fraction of participants stating to be doing 2–5 h per week since implementation of the lockdown measures. **c** Median daily step count was significantly reduced (6777 [IQR 4967–8825] vs. 4829 [IQR 2338–7841], p < 0.01). (D) A significant difference was also found when comparing the selected days (Sunday, 5455 [IQR 2500–8971] vs. 4500 [IQR 1114–8094], Monday, 7378 [IQR 4889–10,000] vs. 4537 [IQR 1541–8228], Tuesday, 7220 [IQR 4767–9998] vs. 4792 [IQR 1782–8396], p < 0.01 for all comparisons)

Daily step count analyses revealed an average daily step count of 6777 [IQR 4967—8825] steps/day, reflecting an active cohort of young adults. After implementation of lockdown, there was a 25% reduction of daily step count (4829 [IQR 2338—7841] steps/day, Fig. 1c). Significant differences were also found when comparing respective days from both periods (Fig. 1d).

In our cohort, implementation of the government lockdown significantly altered physical activity. More than 40% of our subjects reported a relevant decrease in physical activity after lockdown. Due to the uniqueness of the COVID-19 outbreak, these data are difficult to compare. In the published literature, there is no existing data investigating changes in physical activity during pandemics. However, a previous study has shown a significant decrease in physical activity following the catastrophic earthquake and tsunami in Japan in 2011 [3].

In our cohort, about one-third of our subjects reported increased physical activity during the COVID-19 pandemic. This may be related to the fact that Bavaria has not imposed a full-scale lockdown such as those in France, Spain, and Italy. In contrast, the federal states of Germany permitted outside exercise, but only alone, or with other household members. Thus, an impressive number of study subjects even increased physical activity.

In order to better quantify activity levels, we assessed the daily step count on three consecutive days after lockdown, and, in comparison, on three consecutive days before the first patient was diagnosed with COVID-19 in Bavaria. We detected a remarkable reduction of median daily step count after implementation of the lockdown, confirming the results of the self-assessment in the questionnaire. Daily step counts have been established as an overall measure of physical activity and an excellent prognostic value for all-cause mortality in both, healthy individuals, and patients with cardiovascular and pulmonary diseases [4, 5]. Despite the significant decrease of daily step counts, the long-term effect on morbidity and mortality is completely unpredictable at this moment.

The major limitation of our study is that the results do not apply to the physical activity behaviour in elderly individuals or for specific risk groups, for which physical activity is essential for physical and psychical health conditions.

In summary, we were able to show, for the first time, a change in physical activity among young adults during the COVID-19 crisis. Further studies investigating long-term effects of pandemic related changes in physical activity on morbidity and mortality are warranted.

## Data Availability

The data supporting the conclusions of the article is included in the article.
